# New potential binding determinant for hERG channel inhibitors

**DOI:** 10.1038/srep24182

**Published:** 2016-04-12

**Authors:** P. Saxena, E.-M. Zangerl-Plessl, T. Linder, A. Windisch, A. Hohaus, E. Timin, S. Hering, A. Stary-Weinzinger

**Affiliations:** 1 Institute of Pharmacology and Toxicology, University of Vienna, Austria

## Abstract

Human ether-à-go-go related gene (hERG) 1 channels conduct the rapid delayed rectifier K^+^ current (I_Kr_) and are essential for the repolarization of the cardiac action potential. hERG1 inhibition by structurally diverse drugs may lead to life threatening arrhythmia. Putative binding determinants of hERG1 channel blockers include T623, S624 and V625 on the pore helix, and residues G648, Y652 and F656, located on segment S6. We and others have previously hypothesized that additional binding determinants may be located on helix S5, which is in close contact with the S6 segments. In order to test this hypothesis, we performed a detailed investigation combining ionic current measurements with two-microelectrode voltage clamp and molecular modeling techniques. We identified a novel aromatic high affinity binding determinant for blockers located in helix S5, F557, which is equally potent as Y652. Modeling supports a direct interaction with the outer pore helix.

Human ether-à-go-go related gene (hERG) 1 channels conduct the rapid delayed rectifier K^+^ current (I_Kr_) and are essential for regulating the duration of the plateau phase of the cardiac action potential^1^,^2^. Inherited loss-of-function mutations in hERG1 can lead to life threatening torsades de pointes (TdP) arrhythmia^3^, while gain-of-function mutations are associated with short QT syndrome^4^. Most frequently, TdP arrhythmia is an acquired disorder, resulting from “off-target” block of this channel by structurally diverse drugs including antiarrhythmics, antihistamines, antipsychotics and antibiotics^5^. Since this inhibition can lead to sudden cardiac death, several pharmaceuticals such as cisapride or terfenadine were withdrawn from the market, or had their use severely restricted^6^,^7^. Recently, the Cardiac Safety Research Consortium (CSRC) and the Food and Drug Administration (FDA) proposed a new cardiac safety paradigm labelled “Comprehensive *In Vitro* Proarrhythmia Assay” (CiPA). The new CiPA guidelines emphasize the importance of studying pharmacological effects of drugs on three different ion channel types including hERG, Nav1.5 and Cav1.2, which proposed to play an important role in shaping the ventricular action potential^8^. hERG1 blockers might also have beneficial therapeutic potential. During routine preclinical screening for hERG1, new modulators, so-called activators, have been identified. These modulators may have the potential of shortening the action potential duration^9^. Thus, they might be beneficial for the treatment of inherited long QT syndrome.

Great efforts have been directed toward a better understanding of the molecular and structural mechanisms of hERG1 channel drug interactions, including *in vivo*, *in vitro* and *in silico* approaches (for a recent reviews see Durdagi, S. *et al.*^10^ and Vandenberg, J. *et al.*^11^). Substantial progress has been made by identifying amino acids essential for drug block. The majority of hERG inhibitors are interacting with the pore module. This homo tetrameric module consists of an outer S5 helix, a pore helix P1 and an inner helix S6. They include T623, S624 and V625 from the P1, and residues G648, Y652 and F656 located on the helix S6^12^,^13^,^14^,^15^. We and other groups have presented *in silico* modeling studies to provide qualitative^16^,^17^,^18^,^19^ and in some cases quantitative^20^,^21^ insights into drug-channel interactions. However, no consensus about the binding mode(s) has been achieved. It was previously suggested that helix S5, which is in close contact with the S6 segments, might influence drug binding^17^,^18^,^22^. In particular, hydrophobic binding pockets, involving the interface between two subunits and the outer S5 helix, have been discovered as binding site for some small molecule hERG activators^23^,^24^.

According to our validated homology model (hERG-_KvAP-m6_)^22^, F557 on S5 is in close contact with the side chain of Y652 on S6. Thus, we assumed it might be possible for blockers to access lateral side openings, as has been shown recently for sodium channel blockers^25^. In order to test this hypothesis, we combined mutagenesis, voltage clamp analysis and molecular modeling techniques. Thereby, we identified a novel aromatic high affinity binding determinant for hERG blockers in helix S5.

## Results

### Effect of activator binding determinant F557 on pore blockers

**Figure 1:**
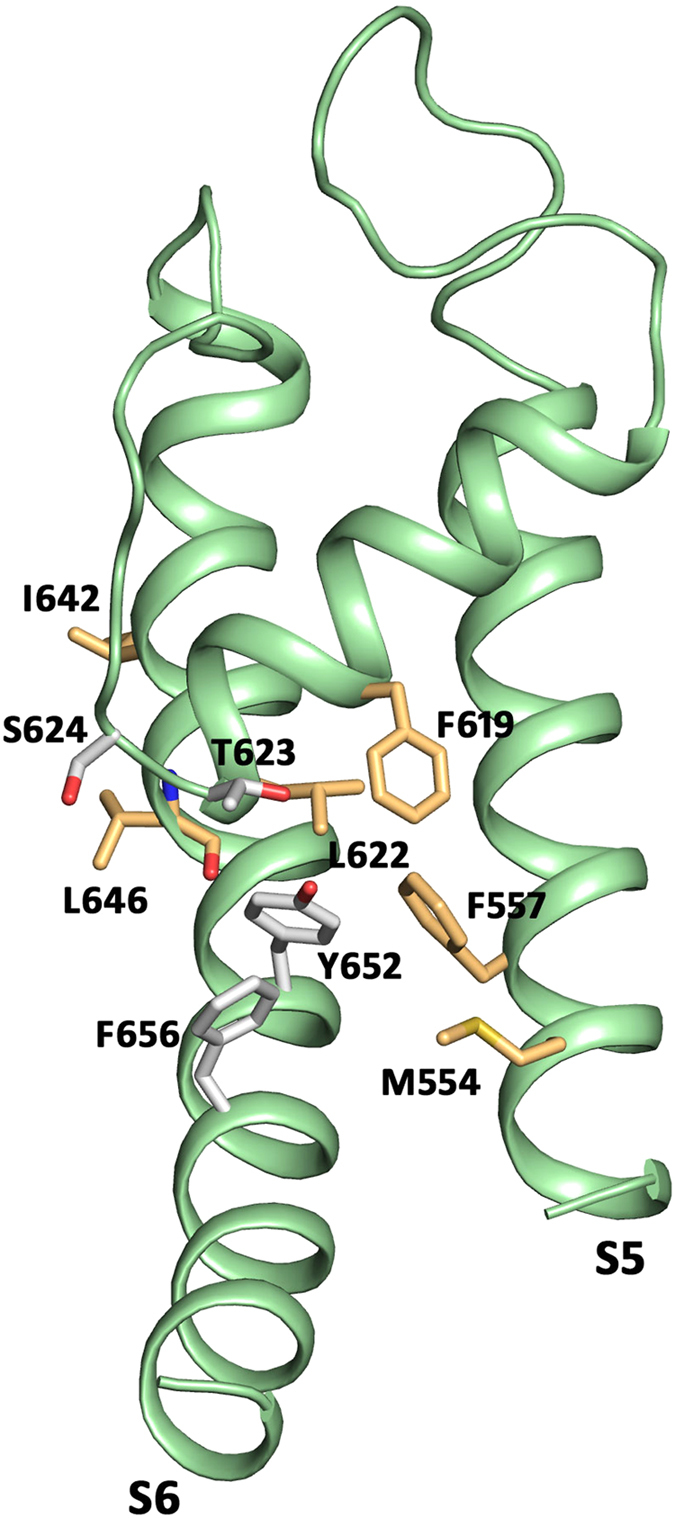
Location of tested residues and known binding determinants T623, S624, Y652 and F656. All residues are represented as sticks in one subunit of the hERG_KvAP-m6_ homology model.

**Figure 2:**
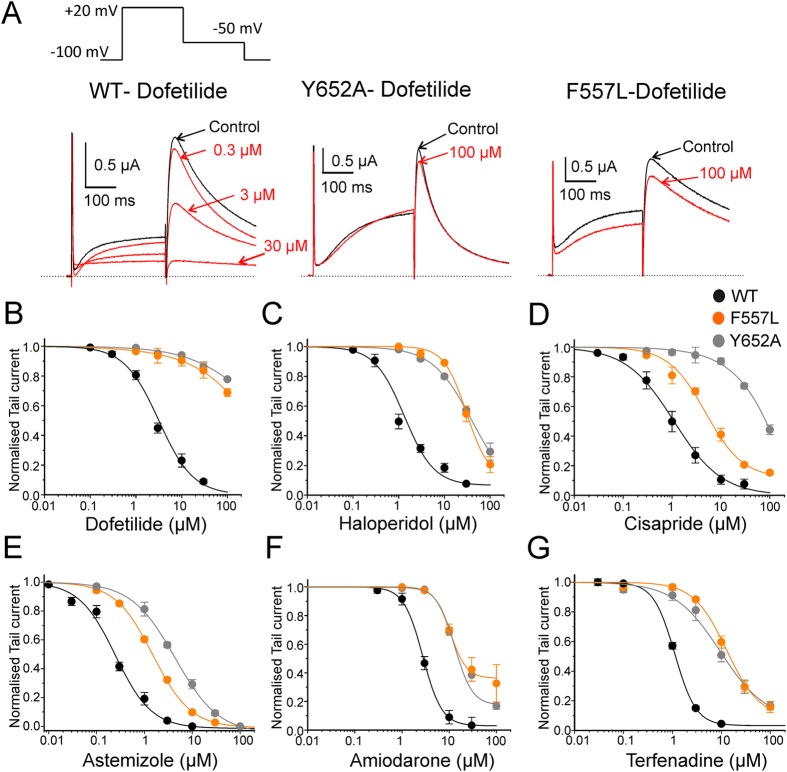
Mutation of F557L alters the sensitivity of hERG channel to drug block. (**A**) Representative current traces of hERG and the indicated mutants in response to the pulse protocol illustrated. (**B–G**) Concentration-response relationships for block of WT, mutants F557L and Y652A channels by dofetilide (**B**) haloperidol (**C**) cisapride (**D**) astemizole (**E**) amiodarone (**F**) and terfenadine (**G**).

To determine whether phenylalanine in position 557 (see Fig. 1 for location) affects hERG inhibition, we introduced the point mutation F557L. This mutation exhibits normal P-type inactivation, as reported previously^23^. Both, the F557L mutant and the wild type hERG (WT) channels were expressed in *Xenopus laevis* oocytes and potassium currents were measured with the two-microelectrode voltage clamp technique. Six “gold standard” hERG blockers (dofetilide, haloperidol, cisapride, astemizole, amiodarone and terfenadine) were used to validate the potential impact of F557. For hERG measurements, the oocytes were clamped at a holding potential of −100 mV and depolarized to +20 mV allowing activation and inactivation. Subsequent recovery from inactivation during a repolarisation to −50 mV induced large tail currents (Fig. 2A). Channel block was estimated from tail current inhibition. Substantial shifts of the concentration response curves of F557L to the right and incomplete hERG inhibition at high concentrations compared to WT (Fig. 2B–G) illustrates strong impairment of channel block. In order to estimate the severity of this effect, we compared concentration response relationships of F557L with mutant Y652A, which is known to efficiently diminish hERG inhibition^12^,^15^. Both mutations induced comparable shifts of the concentration response curves compared to WT channels for all studied blockers. This suggests that F557L on segment S5 is an equally strong molecular determinant of hERG inhibition as the well-established putative binding determinant Y652 (Fig. 2).

For further validation of the impact of residue F557, we estimated the inhibition of currents through WT, F557L and Y652A at concentrations 10 times the WT IC_50_ (=half maximal inhibitory concentration). The corresponding concentrations were 25 μM for dofetilide, 10 μM for haloperidol, 10 μM for cisapride, 3 μM for astemizole, 30 μM for amiodarone and 10 μM for terfenadine. The effects of F557L and Y652A on tail current inhibition by these drug concentrations are illustrated in Supplementary Fig. S1. Channel inhibition in F557L and Y652A by dofetilide, haloperidol, amiodarone and terfenadine was comparable. Parallel IC_50_ shifts to the right (>50 fold increase) of F557L and Y652A were observed in case of dofetilide. For haloperidol, we observed a 23 fold increase in IC_50_ relative to WT with F557L compared to a 31 fold increase in IC_50_ induced by Y652A. The least pronounced (minimum fold) increase in IC_50_ was observed for amiodarone, with 3 fold (F557L) and 5 fold (Y652A) increases, respectively. Similar parallel IC_50_ shift to right was observed for terfenadine with 12 fold (F557L) and 8 fold (Y652A) increase relative to WT. In case of cisapride, a 9 fold increase in IC_50_ was observed with F557L compared to 74 fold increase in IC_50_ with Y652A. Similarly, astemizole showed 6 fold shift in IC_50_ for F557L and 18 fold shift for Y652A. Both cisapride and astemizole showed less pronounced increase in IC_50_ on mutant F557L in comparison to fold increase in IC_50_ by Y652A (See Table 1 for all studied hERG inhibitors). Taken together, F557L shifted the concentration response curves for all studied hERG blockers to the right, ranging from a 4 fold (amiodarone) to more than 50 fold (dofetilide) increase in IC_50_.

### Role of residues surrounding F557 on hERG inhibition

**Figure 3:**
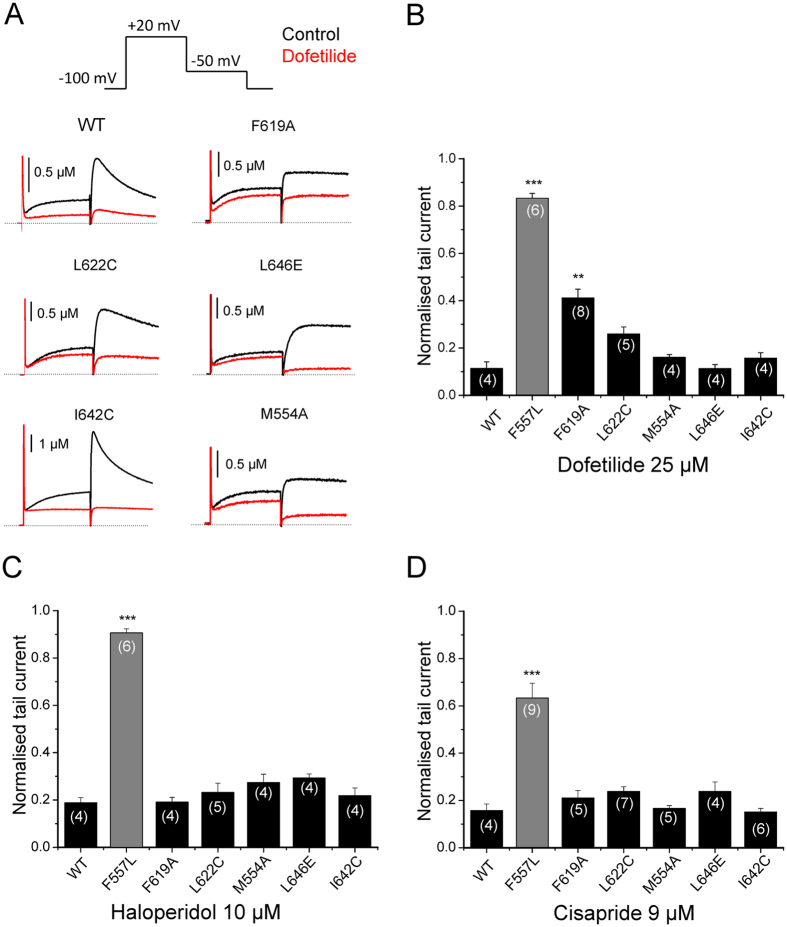
Effects of mutations surrounding F557 on channel inhibition by dofetilide, haloperidol and cisapride. (**A**) Representative currents through the indicated hERG channel mutants in control (black traces) and after steady state block by 25 μM dofetelide was reached (red traces, same voltage protocol as shown in top panel. (**B–D**) Inhibition of currents through the indicated mutants by dofetilide (25 μM), haloperidol (10 μM) and cisapride (9 μM). Data are represented throughout the figure as mean ± SEM. The numbers of experiments (n) are indicated as small insets within the bars. ***P < 0.001, **P < 0.01, one-way ANOVA. The value of 1 indicates no detectable decrease in current by drugs.

F557 was previously identified as potential interaction site for hERG agonists^23^,^24^. It was therefore interesting to investigate if other high affinity agonist binding determinants, located in the vicinity of F557, would also affect channel inhibition. Consequently, we tested residues M554 located on S5, F619 and L622 located on P1, I642 and L646 located on S6, previously identified by Garg *et al.*^23^ (for location of tested residues see Fig. 1). Figure 3A–D illustrates the effects of these mutants on current inhibition by dofetilide, haloperidol and cisapride at concentration 10 times the WT IC_50_. Figure 3A,B shows that the effect of dofetilide on current inhibition was not altered by mutations L622C, M554A, L646E, and I642C. However, a moderate effect on channel inhibition by dofetilide was observed for F619A. As illustrated in Fig. 3C,D none of these mutations significantly affected the channel inhibition by haloperidol and cisapride, respectively.

### Choice of template determines size of lateral pore openings

To address whether residue F557 indeed influences drug block via direct π-π and/or hydrophobic interactions, or if the experimentally observed effects are allosteric, we performed *in silico* modeling studies. Due to the absence of hERG crystal structures, homology modeling techniques were employed to investigate drug binding. Crystal structures of several K^+^ channels have been determined in closed, open and putatively open inactivated states, enabling modeling of hERG in different channel states. Since all x-ray structure templates have low sequence identity to hERG, the correct choice of template/s and alignment is not trivial^22^. In order to minimize shortcomings based on template choice or channel state, we chose to systematically investigate the role of F557 using five different hERG homology models, based on open and/or open inactivated K^+^ channel templates, using our previously published alignments^22^. As can be seen in Supplementary Fig. S2, the choice of K^+^ channel template has a profound influence on the spatial arrangement of the molecular determinants Y652, F656 and F557.

In a first step, we analyzed the size of the lateral openings in the different hERG homology models using the pymol plugin caver 3.0.1^26^. The center of residue G648 in helix S6 of all 4 chains was used as a starting point. The diameter of the narrowest “constriction” in the fenestration pathway of the different hERG models can be found in Table 2. As shown in Supplementary Fig. S3, the size of the lateral pore openings varies, depending on the choice of template. Lateral pore openings towards S5, which are presumably large enough for drug interactions, are found in three models: the open state model based on hERG_KvAP-m6_^22^, the KcsA-inactivation based model hERG_KcsA-I_^27^,^28^ and the MthK based model hERG_MthK-L_^29^. Consequently, only in these three models possible direct drug interactions with F557 in helix S5 were investigated; docking in all other models did not lead to any acceptable poses, despite allowing free rotation of all binding aromatics (data not shown).

### Modeling suggests direct interaction of high affinity hERG blockers with F557

**Figure 4:**
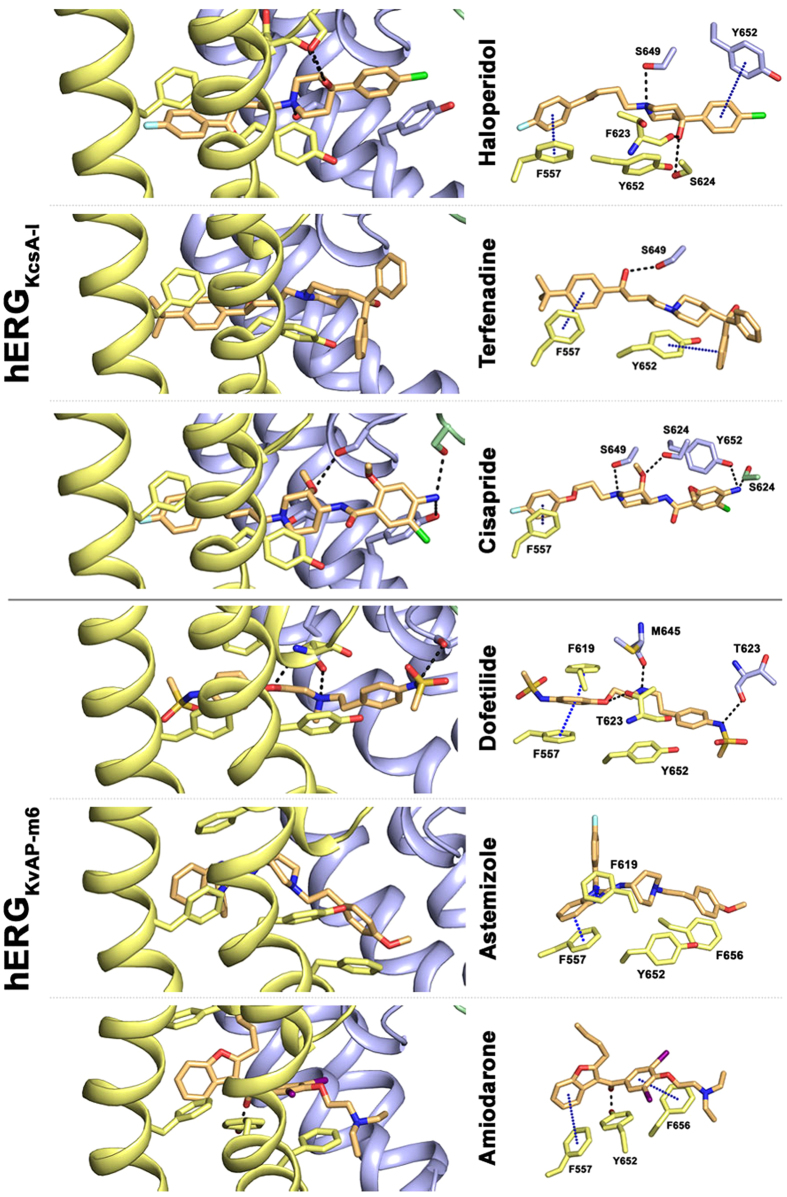
Docking poses revealing aromatic interactions with residue F557. Left side: side view of two neighboring subunits colored in yellow and blue. Right side: top view of hERG blockers and interacting residues, presented as sticks. π-π interactions between aromatic rings are indicated by blue dots (cutoff distances: sandwich-shaped = 5Å; t-shaped = 6.5 Å; parallel displaced = 5.5 Å)^56^.

To investigate, if π-π or cation-π interactions between high affinity blockers and residue F557 are possible, we performed docking simulations. Figure 4 summarizes the best docking poses (highest binding affinity from Chemscore, summarized in Supplementary Table S1) obtained for each compound. In case of amiodarone, astemizole and dofetilide, the highest score poses were obtained with model hERG_KvAP-m6_. For cisapride, haloperidol and terfenadine, the highest ranked poses were obtained in hERG_KcsA-I_.

Generally, direct π-π interactions between drugs and the aromatic side chain of F557 are possible for all drugs. These direct interactions influence the positioning of the blockers in the central cavity. Binding poses enabling π-π interactions with F557 are characterized by protrusion into the lateral openings between two neighboring subunits, below the selectivity filter. For all studied drugs, extensive ring stacking or hydrophobic interactions with several aromatic side chains including Y652, F656 and F557 were observed. In case of haloperidol, terfenadine, cisapride and dofetilide, polar interactions with T623 and/or S624 are predicted. Further, the polarizable nitrogen of the investigated blockers in cisapride and terfenadine is positioned right below the pore helix, suggesting that the helix dipole charges contribute to binding (see Fig. 4).

## Discussion

It is widely accepted that block of hERG K^+^ channels by structurally diverse molecules is mediated by two aromatic side chains Y652 and F656^12^. Mutation of either residue to alanine dramatically reduces drug potency. Modeling studies suggest direct interaction of drugs with these residues^16^,^18^,^30^. According to our recent hERG model^22^, residue F557 in helix S5 is in close proximity with Y652 (S6). This residue was identified as crucial molecular determinant for the effect of hERG activator ICA-105574^23^. In order to test the hypothesis that determinants of hERG activators might interact with hERG blockers, we mutated F557 and analysed the inhibition of potassium currents through mutant F557L by six “gold standard” hERG blockers dofetilide, haloperidol, terfenadine, astemizole, cisapride and amiodarone. Our study reveals that mutation F557L dramatically decreases current inhibition. To our surprise, F557L reduced the drug potency to the same extent as the well-studied mutation Y652A (Fig. 2). We further investigated if other residues in close proximity of F557 (see Fig. 1), such as F619, L622, M554, L646 and I642 also influence channel inhibition. However, except for F619A, which had a moderate effect on channel inhibition by dofetilide, none of the studied mutations significantly affected channel inhibition by the tested drugs (Fig. 3).

To investigate the role of F557 in more detail, we performed modeling studies to elucidate potential interactions between F557 in helix S5 and high affinity blockers tested in our study. Docking studies with different open or open/inactivated hERG models (Supplementary Figs S2 and S3) suggest that all studied blockers bind in the cavity, below the selectivity filter, partially protruding into lateral, hydrophobic pore openings (Fig. 4). These openings, also known as fenestrations, have first been identified in bacterial sodium channels^31^,^32^. Interestingly, the novel binding modes for hERG blockers proposed in this study resembles recently reported binding sites of brominated sodium channel blockers in the bacterial sodium channel NavMs crystal structure^25^. A recent x-ray analysis with quaternary ammonium blockers in KcsA, suggests that fenestrations in this potassium channel are only accessible, if rotameric changes of the aromatic side chain of residue F103 occur^33^.

Interestingly, reshaping of fenestrations has been suggested to play an important role in slow inactivation in bacterial sodium channels^32^. Thus, it is conceivable that binding of small molecules to the hydrophobic side openings affects the affinity of drugs to the inactivated state. This is further supported by recent studies, revealing the binding site for a small molecule activator in hERG at this side, which strongly attenuates inactivation^23^. The mechanisms, how inactivation influences drug affinity in different ion channels, is still poorly understood. Based on our modeling with different K^+^ channel structure templates, one might speculate that the geometry of the hydrophobic side pockets depends on the geometry of the helices and the rotameric state(s) of the aromatic side chains, which are linked to the channel state. Interestingly, previous modeling studies on hERG observed transiently occurring hydrophobic openings between subunits, exposing the central cavity to the hydrophobic core of the lipid membrane^17^,^18^. Further, x-ray and MD studies on KcsA clearly revealed a structural link between the rotameric state of F103 (located at the entrance of the fenestration) in KcsA, the intracellular gate and the selectivity filter^27^,^34^.

Taken together, all these studies suggest that binding of drugs to hydrophobic side pockets might be quite common for cation channels, and simultaneously, may play a role in inactivation gating. However, this issue clearly warrants further research.

The novel binding mode for high affinity blockers proposed in this study, is in good agreement with most published experimental data available for hERG blockers^12^,^14^,^15^,^35^,^36^,^37^,^38^,^39^,^40^,^41^. However, tandem dimer mutant studies^30^ for cisapride and terfenadine reporting on the number of aromatic interactions are not fully consistent with the proposed binding mode for these drugs from our study. In particular, the arrangement of Y652 and F656 from one subunit and the Y652 side chain from an adjacent subunit are not seen in any poses, where F557 directly interacts with the drug. Such poses are only possible in the conventional binding mode. A plausible explanation for this discrepancy is the existence of several binding modes for hERG blockers. This would also explain, why so many different hERG binding modes have been predicted in numerous previous studies^16^,^18^,^21^,^42^,^43^,^44^,^45^. The heterogeneous nature of drug binding in hERG might further explain why so many structurally diverse compounds can block this channel. Additionally, it is more and more appreciated that small molecule interactions with ion channels can occur at many sites^46^,^47^,^48^. Alternatively, we cannot exclude that F557 does not contribute directly to drug binding but, instead, modulates channel block allosterically. However, our simulation studies strongly support a direct interaction.

Summarizing, our study reveals a putative novel high affinity binding determinant for hERG blockers. Further, we propose the existence of a novel hydrophobic binding site, at the fenestrations, additionally to the conventional binding site in the central cavity.

## Materials and Methods

### Oocyte electrophysiology

cDNAs of hERG (accession number NP000229) and constructs F557L, F619A, L622C, M554A, L646E, I642C were kindly provided by MC Sanguinetti (University of Utah, UT, USA) and JI Vandenberg (Molecular Cardiology and Biophysics Division, Victor Chang Cardiac Research Institute, Darlinghurst, New South Wales). Synthesis of capped runoff complementary ribonucleic acid (cRNA) transcripts from linearized complementary deoxyribonucleic acid (cDNA) templates and injection of cRNA were performed as described previously^49^. Oocytes from the South African clawed frog, *Xenopus laevis*, (NASCO, Fort Atkinson, WI, USA) were prepared as follows: After 15 min exposure of female *Xenopus laevis* to the anaesthetic (0.2% solution of MS-222; the methane sulfonate salt of 3-aminobenzoic acid ethyl ester; Sigma), parts of the ovary tissue were surgically removed. Oocytes were defolliculated by enzymatical treatment with 2 mg/mL collagenase type 1 A (Sigma) and further by mechanical removal of follicular layer using forceps. Stage V–VI oocytes were selected and injected with the WT hERG-encoding cRNA. Injected oocytes were stored at 18 °C in ND96 bath solution (96 mM sodium chloride, 2 mM potassium chloride, 1 mM magnesium chloride, 1.8 mM calcium chloride, 5 mM HEPES; pH 7.4, titrated with NaOH) containing 1% penicillin-streptomycin solution. All chemicals used were purchased from Sigma- Aldrich Chemie GmbH, Taufkirchen, Germany.

Currents through hERG channels were studied 1–2 days after microinjection of the cRNA using the two-microelectrode voltage clamp technique. ND96 was used as an extracellular recording solution. Voltage-recording and current-injecting microelectrodes were filled with 3 M KCl, and had resistances between 0.3 and 2 MΩ. Endogenous currents (estimated in oocytes injected with water) did not exceed 0.15 *μ*A. Currents >5 *μ*A were discarded to minimize voltage clamp errors. A precondition for all measurements was the achievement of stable peak current amplitudes over periods of 10 min after an initial run-up period. All drugs were applied by means of semi-fast perfusion system^50^. Ionic currents were recorded with a Turbo Tec 03X Amplifier (npi electronic, GmbH, Tamm, Germany) and digitized with a Digidata 1322A digitizer (Axon Instruments Inc., Union City, CA, USA). The pClamp software package version 9.2 (Axon Instruments Inc.) was used for data acquisition. Microcal Origin 7.0 was employed for analysis and curve fitting. Currents through wild type hERG channels and channel mutants were studied at a holding potential of −100 mV using a 2 step voltage protocol. A conditioning 300 ms depolarizing step to +20 mV was followed by a 300 ms repolarization to −50 mV. Repolarisation induced large tail currents. Drug effects were analyzed after a 15–20 minute “run-up” phase was completed. Drug effects were estimated from peak tail current inhibition at −50 mV after steady state was reached. Tail current concentration–inhibition curves were fitted using the Hill equation (see equation (1)).


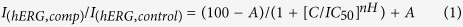


In the hill equation, *IC*_*50*_ is the concentration at which hERG inhibition is half-maximal, *C* is the applied drug concentration in μM, *A* is the fraction of hERG current that is not blocked and *nH* is the Hill coefficient. Data points represent means ± s.e. from at least three oocytes from >2 batches; Statistical significance was calculated using a one-way ANOVA followed by a bonferroni’s multiple comparison test **p < 0.01 and ***p < 0.001, respectively. All the studied compounds were purchased from Sigma Aldrich. All compounds were first dissolved in dimethyl sulphoxide (DMSO) to prepare a 10 mM stock solution and stored at −20 °C. Drug stocks were diluted to the required concentration in extracellular solution on the day of each experiment. The maximal DMSO concentration in the bath (0.1%) did not affect hERG currents.

### Ethical statement

All experiments involving animals were approved by the Austrian Animal Experimentation Ethics Board in accordance with the European convention for the protection of vertebrate animals used for experimental and other scientific purposes ETS no. 123, which is in line with the EU Directive 2010/63/EU (GZ 66.006/0019-C/GT/2007). The methods were carried out in accordance with the approved guidelines.

### Homology modeling

Homology models of the open and inactivated hERG pore domain, based on different K^+^ channel templates, were generated with Modeller 9v12. Templates include the pore domain of the KcsA crystal structure (Protein Data Bank (pdb) ID 3F5W^27^,^51^), the Kv1.2 structure (pdb ID: 3LUT^52^) and the MthK x-ray structures (pdb ID: 1LNQ^29^ and 3LDD^53^). Modeling details, including coordinates for the KvAP based open conformation, have been described previously^22^ (model m6). Alignments of the models are described in Stary *et al.*^22^ (see Fig. 1 in ref. 22).

### Analysis of fenestrations

Caver parameters were used as previously described by Kaczmarski & Corry^54^ (Probe_radius = 0.8 Å; Shell_radius = 15 Å; Shell_depth = 15 Å; Clustering_threshold = 10 Å; Number_of_approximating_balls = 12). The starting point was selected using the center of the G648 residue in the S6 of all 4 chains.

### Drug docking and parametrization

Docking was performed using the program Gold 4.0.1 and the implemented Gold scoring function^55^. hERG homology models in the open state, based on the KvAP template termed “hERG-_KvAP-m6_”^22^, a high resolution MthK structure in open conformation (3LDD^53^) termed “hERG-_MthK-O_”, the high resolution structure of Kv1.2 (3LUT^52^), termed “hERG-_Kv1.2_” and two putatively inactivated state models based on the KcsA template 3F5W^27^,^51^, termed “hERG-_KcsA-I_” and the low resolution x-ray MthK structure 1LNQ^29^ termed “hERG-_MthK-I_” were used for docking^22^. The binding site in all models was defined by setting Y652 of one SU as the center and the radius to 10 Å. This binding site thereby included the reported binding determinants Y652, F656, T623, S624 and the putative interacting aromatic amino acids F557 and F619. The rotameric state of these amino acids was set to flexible, allowing free sampling of side chain conformations. For all drugs, the central nitrogen was protonated and used in their charged form. The 20 best-ranked poses of each drug docking run were visually inspected.

## Additional Information

**How to cite this article**: Saxena, P. *et al.* New potential binding determinant for hERG channel inhibitors. *Sci. Rep.* **6**, 24182; doi: 10.1038/srep24182 (2016).

## Supplementary Material

Supplementary Information

## Figures and Tables

**Table 1:** Summary of IC_50_ values and hill coefficients (nH) determined from concentration-response relationships for all studied blockers.

Name	WT		Y652A-hERG			F557L-hERG		
	IC_50_ (μM)	nH	IC_50_(μM)/% block @100 μM	nH	∆IC_50_	IC_50_(μM)/% block @100 μM	nH	∆IC_50_
Dofetilide	2.5 ± 0.2 n = 4	1.3 ± 0.2	23% n = 5	0.6 ± 0.2	>50	31% n = 7	0.5 ± 0.4	>50
Terfenadine	1.1 ± 0.04 n = 6	2 ± 0.2	9.3 ± 2.3 n = 4	1.03 ± 0.2	8.4	12.8 ± 2.3 n = 8	1.3 ± 0.1	11.6
Astemizole	0.25 ± 0.02 n = 6	1.1 ± 0.1	4.4 ± 0.3 n = 5	0.9 ± 0.1	17.6	1.6 ± 0.2 n = 6	1.1 ± 0.1	6.4
Cisapride	1.1 ± 0.1 n = 5	0.9 ± 0.03	81.4 ± 8.1 n = 4	1.03 ± 0.1	74	10.1 ± 0.6 n = 7	0.7 ± 0.03	9.2
Haloperidol	1.3 ± 0.1 n = 4	1.3 ± 0.1	40.1 ± 1.9 n = 4	1 ± 0.04	31.4	30.3 ± 7.5 n = 4	1.7 ± 0.3	23.3
Amiodarone	2.7 ± 0.3 n = 5	2.1 ± 0.4	13.5 ± 1.05 n = 5	2.3 ± 0.2	5	9.02 ± 1.05 n = 5	3.1 ± 0.2	3.34

(n = number of experiments).

**Table 2:** Fenestration pathway diameters and the residues contributing to the narrowest point of different hERG models.

Model	Diameter of closest fenestration point	Residues contributing to the narrowest point
hERG-_KvAP-m6_	1.98 Å	1^st^ Subunit: F557, F619
		2^nd^ Subunit: L646
hERG-_MthK–I_	1.71 Å	1^st^ Subunit: F619, M651, I655
		2^nd^ Subunit: L646, M645
hERG-_KcsA–I_	1.27 Å	1^st^ Subunit: T623, Y652
		2^nd^ Subunit: M645, S649
hERG-_Kv1.2_	1.03 Å	1^st^ Subunit: F557, F619
		2^nd^ Subunit: L646
hERG-_MthK-O_	No path could be found at the discussed lateral fenestration site	
