# Supplementary material for: New potential binding determinant for hERG channel inhibitors
Source: Sci Rep. 2016 Apr 12;6:24182. doi: 10.1038/srep24182 (PMC4828713; doi:10.1038/srep24182)
Supplement: Supplementary Information [file srep24182-s1.doc]

Supplemental material

**New potential binding determinant for hERG channel inhibitors**

Saxena P^1^, Zangerl-Plessl E-M^1^, Linder T^1^, Windisch A, Hohaus A, Timin E, Hering S, Stary-Weinzinger A*


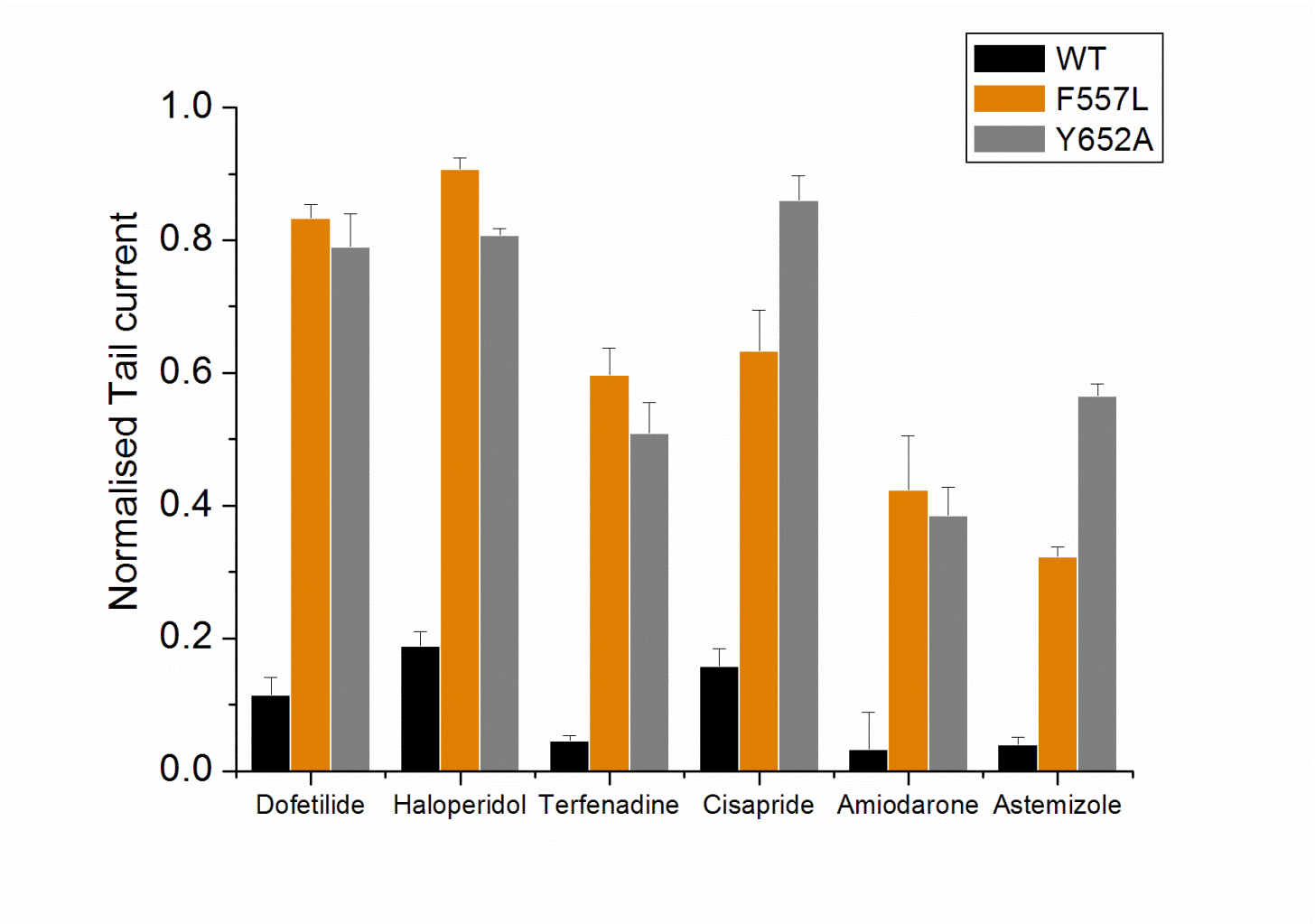


**Supplementary Figure S1. Effect on WT, mutant F557L and Y652A hERG channels block by hERG inhibitors.**

Normalized current (I_drug_/ I_control_) measured after steady state block by concentration 10 times IC_50_ of each drug. n = 4-8 (mean, error bar ±SEM). A value of 1 indicates no detectable decrease in peak tail current by the drug.


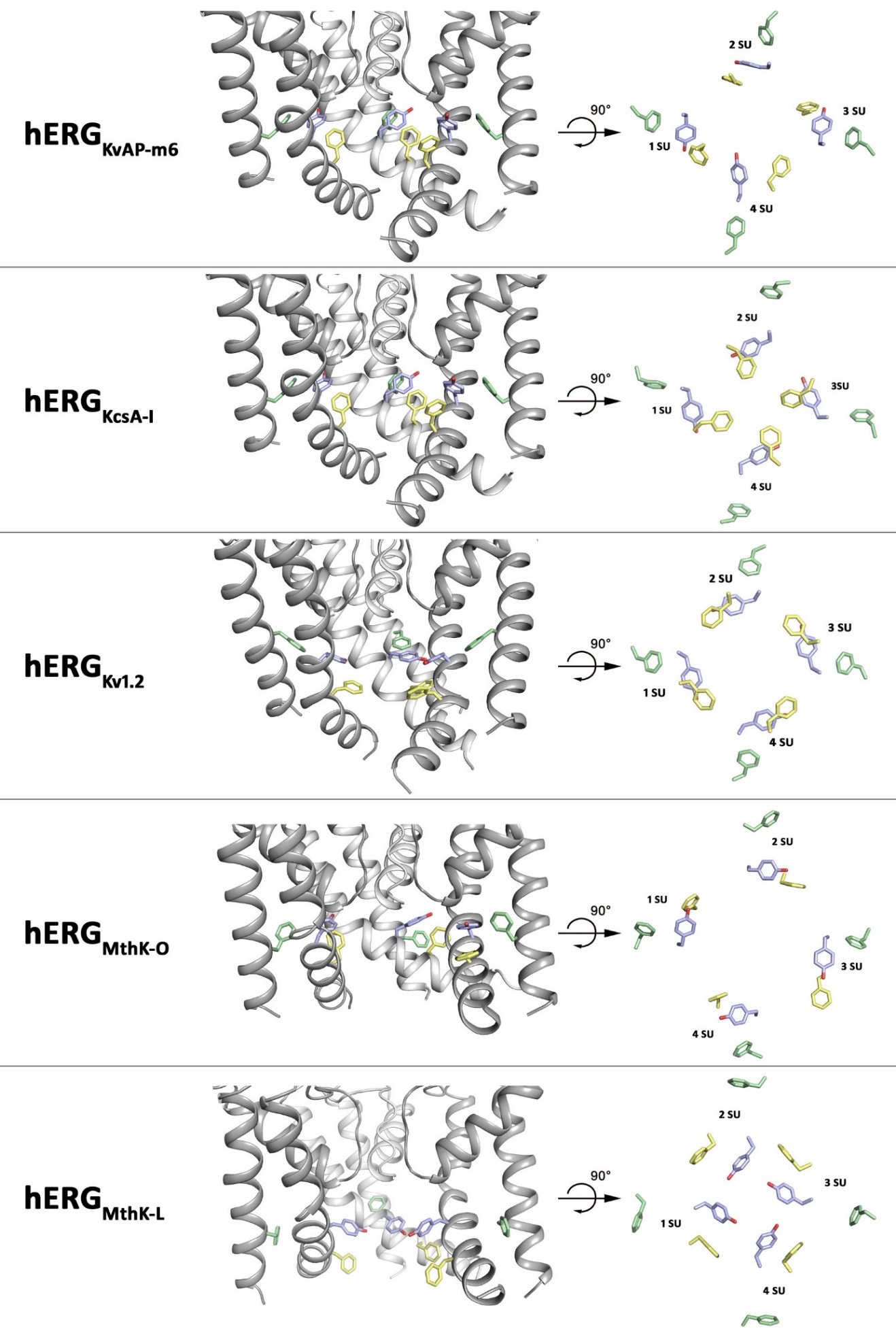


**Supplementary Figure S2. Overview of hERG models investigated in this study.**

Left side: side view of hERG homology models, only 3 subunits are shown, the fourth one is omitted for clarity reasons. F557, Y652 and F656 are shown as sticks and colored in yellow, blue and green, respectively. Right side: top view showing F557, Y652 and F656 positions of all four SU colored in green, blue and yellow, respectively.


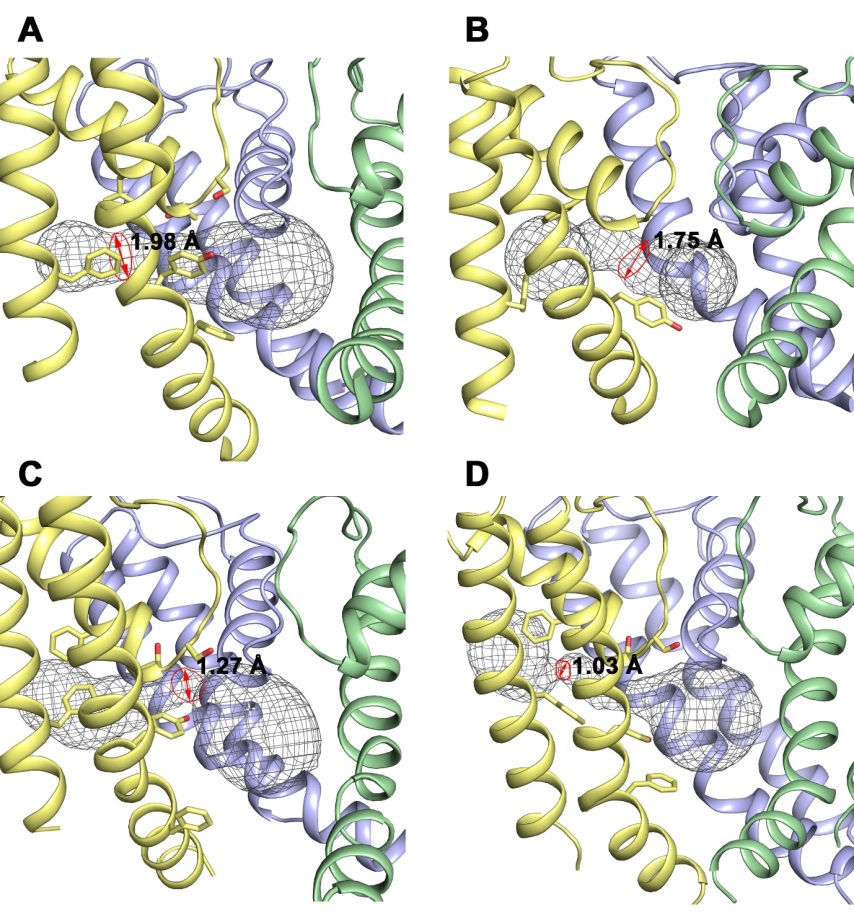


**Supplementary Figure S3. Caver results in different hERG models.**

Lateral pore openings towards the lipids in different hERG models between two chains (yellow and blue) calculated using the caver 3.0.1 plugin in pymol (illustrated as grey mesh). Each closest fenestration diameter is highlighted in red. (A) hERG_KvAP-m6_; (B) hERG_MthK-L_; (C) hERG_KcsA-I_; (D) hERG_Kv1.2_. For the hERG_MthK-O_ model, caver could not find any fenestration pathway between the two adjacent chains.

| **Blocker** | **Homology model** | **Chemscore conventional binding mode**  **[∆Gbind kJ/mol]** | **Chemscore F557 binding mode**  **[∆Gbind kJ/mol]** |
| --- | --- | --- | --- |
| Amiodarone | hERG_KvAP-m6_ | -33.00 | -46.24 |
| Astemizole | hERG_KvAP-m6_ | -29.76 | -44.76 |
| Cisapride | hERG_KcsA-I_ | -30.86 | -32.93 |
| Dofetilide | hERG_KvAP-m6_ | -30.00 | -39.87 |
| Haloperidol | hERG_KcsA-I_ | -27.82 | -30.63 |
| Terfenadine | hERG_KcsA-I_ | -35.21 | -41.13 |

**Supplementary Table S1. Best docking Chemscores for each studied hERG blocker.**
